# Development of Antibiotic Resistance during Simulated Treatment of *Pseudomonas aeruginosa* in Chemostats

**DOI:** 10.1371/journal.pone.0149310

**Published:** 2016-02-12

**Authors:** Yanfang Feng, Caspar J. Hodiamont, Reinier M. van Hest, Stanley Brul, Constance Schultsz, Benno H. ter Kuile

**Affiliations:** 1 Department of Molecular Biology and Microbial Food Safety, Swammerdam Institute of Life Sciences, University of Amsterdam, Amsterdam, The Netherlands; 2 Department of Medical Microbiology, Academic Medical Center, Amsterdam, the Netherlands; 3 Department of Hospital Pharmacy & Clinical Pharmacology, Academic Medical Center, Amsterdam, the Netherlands; 4 Department of Global Health-Amsterdam Institute for Global Health and Development, Academic Medical Center, Amsterdam, The Netherlands; 5 Office for Risk Assessment and Research Coordination Netherlands Food and Consumer Product Safety Authority, Utrecht, The Netherlands; University of Cambridge, UNITED KINGDOM

## Abstract

During treatment of infections with antibiotics in critically ill patients in the intensive care resistance often develops. This study aims to establish whether under those conditions this resistance can develop *de novo* or that genetic exchange between bacteria is by necessity involved. Chemostat cultures of *Pseudomonas aeruginosa* were exposed to treatment regimes with ceftazidime and meropenem that simulated conditions expected in patient plasma. Development of antibiotic resistance was monitored and mutations in resistance genes were searched for by sequencing PCR products. Even at the highest concentrations that can be expected in patients, sufficient bacteria survived in clumps of filamentous cells to recover and grow out after 3 to 5 days. At the end of a 7 days simulated treatment, the minimal inhibitory concentration (MIC) had increased by a factor between 10 and 10,000 depending on the antibiotic and the treatment protocol. The fitness costs of resistance were minimal. In the resistant strains, only three mutations were observed in genes associated with beta-lactam resistance. The development of resistance often observed during patient treatment can be explained by *de novo* acquisition of resistance and genetic exchange of resistance genes is not by necessity involved. As far as conclusions based on an *in vitro* study using *P*. *aeruginosa* and only two antibiotics can be generalized, it seems that development of resistance can be minimized by treating with antibiotics in the highest concentration the patient can endure for the shortest time needed to eliminate the infection.

## Introduction

Antimicrobial treatment of critically ill patients in the intensive care is often complicated by antimicrobial resistance (AMR) development of the targeted microorganisms, even though these were susceptible at the start of treatment [1]. The acquired resistance complicates any further antimicrobial treatment that might be needed and can endanger the health of the patient in the case of recurring infections and should therefore be prevented as much as possible. Conversely, timely and appropriate antibiotic therapy significantly reduces mortality in septic patients on ICU’s [2–4] and resistance decreases the chance of appropriate empirical therapy.

Rational design of strategies to prevent development of resistance require a thorough understanding of the processes causing it. Antibiotic resistance can be acquired by transfer of genetic information between bacteria at the infection site, or it can develop *de novo* through genetic mutations, as a result of exposure to the drug [5,6]. Alternatively, it is conceivable that the resistant variants of the target pathogen were present before start of the treatment, but due to extremely low prevalence did not influence the measured minimal inhibitory concentration (MIC). For optimization of treatment protocols aimed at eliminating an infection successfully without causing emergence of resistance it is important to distinguish between these scenarios. Due to the complexity of all potential and actual interactions between microbes at an infection site, it is not possible to ascertain with certainty the occurrence of *de novo* development of resistance in patients during treatment. Simulation of the treatment in chemostats, however, can provide the necessary controlled and reproducible conditions.

A generally accepted principle for treatment protocols to prevent development of resistance is to have the antibiotic concentration exceed the mutant prevention concentration (MPC) for as much of the treatment period as possible [7]. Treatment principles are based on the average susceptibility of the targeted microbes. However, there is considerable variability between individual bacterial cells and microbes possess effective strategies to rapidly increase their resistance against antibiotics [8,9]. As a result, treatment principles that are based on the functional average cannot exclude the possibility of a few cells surviving and becoming resistant. Though some general guidelines for antibiotic stewardship have been formulated [10], the actual influence of different treatment protocols on the likelihood that resistant cells will emerge is poorly understood [11]. This study addresses the question whether under conditions mimicking those at an infection site during antibiotic treatment, pathogens can survive and emerge resistant to the antibiotic that was applied. *Pseudomonas aeruginosa* was chosen as model organism because it is a major nosocomial pathogen associated with high mortality rates among critically-ill patients [12]. Two commonly used drugs to treat *P*. *aeruginosa* infections, ceftazidime and meropenem, were selected to represent the third-generation cephalosporins with anti-*Pseudomonas* activity, and the carbapenem class of antibiotics, respectively.

## Materials and Methods

### Selection of drug level exposure to ceftazidime and meropenem

Ceftazidime and meropenem concentration-time curves were computer-simulated following clinically used dosing regimens for ceftazidime (a 1000 mg iv loading dose followed by a continuous infusion of 3000 mg over 24 h) and meropenem (a 1000 mg iv bolus infusion administered over 30 minutes three times per day). The simulations were performed on the basis of published population pharmacokinetic models for ceftazidime and meropenem in critically ill patients. These models also account for inter-individual variability in drug exposure [13,14]. One thousand simulations per drug were performed, resulting in a range of concentration-time profiles representative for the critically ill patient population treated according to the applied dosing regimens. From this range, the 5, 50 and 95 percentile of the concentration-time profiles of ceftazidime and meropenem were selected to be mimicked in the chemostat. The 50 percentile was chosen to represent the exposure in a typical critically ill patient, while the 5 and 95 percentile were chosen to illustrate the inter-individual variability in drug exposure within the population. Simulations were performed with nonlinear mixed effects modeling (NONMEM) software package, (version 7.2, ICON plc, Dublin, Ireland).

### Bacterial strain, Antibiotics, Growth medium and culture conditions

The strain used throughout the study was the antibiotic-susceptible wild type strain *Pseudomonas aeruginosa* ATCC27853. Cultures were grown at 37°C in cation-adjusted Mueller Hinton Broth (Sigma-Aldrich), autoclaved at 115°C for 10 minutes. Continuous cultures were performed in Sixfors fermenter vessels (Infors AG, Bottingen, Switzerland) with a working volume of 250 mL, air flow 0.1 l/min, at 37°C and stirred at 250 rpm. In the absence of antibiotics cell density was approximately 10^9^ cells/ml. The pH was regulated at 7.0 by automatically adding sterile 2 N NaOH. Samples were taken at exactly 24 h intervals for a variety of parameters, such as optical density (OD_595_), MIC and maximal growth rate (μ_max_) measurement, bacterial cell morphological observation, bacterial cell counts and sequencing of resistance genes. Steady state was assumed when after a minimum of 5 volume changes all culture parameters had reached constant values. A dilution rate of 0.3^-h^ was chosen to mimic the submaximal growth rate that pathogens can be expected to have at an infection site. To determine maximum growth rates (μ_max_) the growth of batch cultures was followed for 23 hours by measuring the optical density at 595 nm. The μ_max_ was calculated based on the averaged growth rates during exponential phase of 4 independent replicates.

The correct antibiotic concentration-time profiles in the chemostat were maintained by computer controlled continuous infusion of ceftazidime and programmed interval pumping of meropenem. Stock solutions of ceftazidime (Fresenius Kabi) and meropenem (Fresenius Kabi) were prepared freshly for every experiment by dissolving the drugs in water and filter sterilizing (0.2 μm). To allow for continuous infusion in the culture, ceftazidime was added to the culture medium stock bottle which was kept on ice throughout experiments, and changed once in two days. The stability of ceftazidime in culture medium kept on ice was tested and no significant degradation was seen over a period of three days. Meropenem is unstable even at low temperature [15,16]. Therefore, the experimental drug concentration was maintained by computer controlled pumping of stock solutions kept on ice for a maximum of 8 hours.

### Minimum inhibitory concentration (MIC) and mutant prevention concentration (MPC)

MIC was measured by following growth in 96-well plates as described previously [17]. The antibiotic concentrations ranged from 0.06 mg/l to 1,024 mg/l. All measurements were performed in duplicates which had identical results in all measurements. The MIC was defined as the minimal concentration of antibiotic that limited growth to an OD_595_ of 0.2 or less after 23 h. The starting OD was 0.05.

MPC was determined by inoculating >10^10^ cells on antibiotic containing Mueller-Hinton agar plates (Sigma-Aldrich). The reported MPC is the lowest concentration that showed no growth after 48 hours at 37°C. All tests were performed in four replicates.

### Cell density, Cell counting and morphological observation

The cell density was measured spectrophotometrically at a wave length of 595 nm. Cell morphology was observed microscopically using a light microscope at 400X. The total cell number of intact bacteria was determined by colony counts of bacteria grown on antibiotic-free agar plates after appropriate dilutions. To establish the resistant fraction, equal volumes were spread on LB agar plates containing antibiotics. The drug concentrations were 8 mg/l, 24 mg/l and 48 mg/l for ceftazidime, representing the 5, 50 and 95 percentile steady-state concentrations resulting from the computer simulation. For meropenem the 5, 50 and 95 percentile trough concentrations were 0.6 mg/l, 5 mg/l and 15 mg/l respectively. The number of colonies observed after overnight incubation on plates containing a specific antibiotic concentration was used as measure for the number of cells being able to survive at this concentration. This number was divided by total cell count on plates without antibiotics to determine the resistant fraction at that concentration.

### Amplification and sequencing of resistance genes

Mutational changes that are likely to contribute to resistance were identified in a separate study by whole genome sequencing of cultures that had been made resistant by exposure to step-wise increasing concentrations of ceftazidime or meropenem (manuscript in preparation). Such mutations were found in the following genes in ceftazidime exposed cells: *ampD*, *dacB*, *hfq*, and *yerD* and in cells adapted to meropenem: *oprD*, *mexR* and *mexB*. PCR products of four colonies were sequenced to detect the presence of mutations in these genes related to meropenem resistance, but fourteen in the case of ceftazidime, because these sometimes occurred in low frequency. The primers used for PCR to amplify the relevant regions are given in Table 1.

**Table 1:** Primers used for PCR reactions to amplify the relevant regions of the indicated genes.

Gene	Oligonucleotide sequence
*ampD*	Forward 5’ GTAGACCACCACCAGAAG 3’
	Reverse 5’ AATACCTTTCCTCGACGC 3’
*dacB*	Forward 5’ ATCGGGCCTGGAGAAT 3’
	Reverse 5’ TTCGCGTGATGTCCGT 3’
*hfq*	Forward 5’ CCCTTCCAGATGCACCA 3’
	Reverse 5’ TTGTCCGTCTGTTTCCG 3’
*yerD*	Forward 5’ GACATGAAAAAGCCGGAG 3’
	Reverse 5’ CGAAGAAGGTGACTACCA 3’
*oprD*	Forward 5’ CTGCGTGCTATAAGTTAG 3’
	Reverse 5’ CTACGCCCTTCCTTTATA 3’
*mexR*	Forward 5’ AAGCGGATACCTGAAACG 3’
	Reverse 5’ AAGCCTCGCGTGAAAACA 3’
*mexB*	Forward 5’ TCGAGGTGAAGACCGT 3’
	Reverse 5’ TGGTAGTCGGGGATCA 3’

### Statistical analysis

In all experiments duplicate measurements were performed for most parameters, with the notable exception of cell counts, as this parameter showed noticeable variation between replicates and was therefore carried out in fourfold. Still, only rarely the difference between the highest and lowest value exceeded 10%. To ascertain reproducibility all experiments were repeated, with invariably an almost identical outcome. Of all experiments only the first version is reported, as averaging values would not be correct, since each datapoint depends on the preceding and therefore replicates are not independent measurements. Including both experiments in the graphs would result in an uninterpretable figure. As differences in values of minimally a factor of 10, but up to 10.000, were measured with a precision of a factor of 2, P values always were less than 0.001, making statistical analysis irrelevant.

## Results

**Fig 1:**
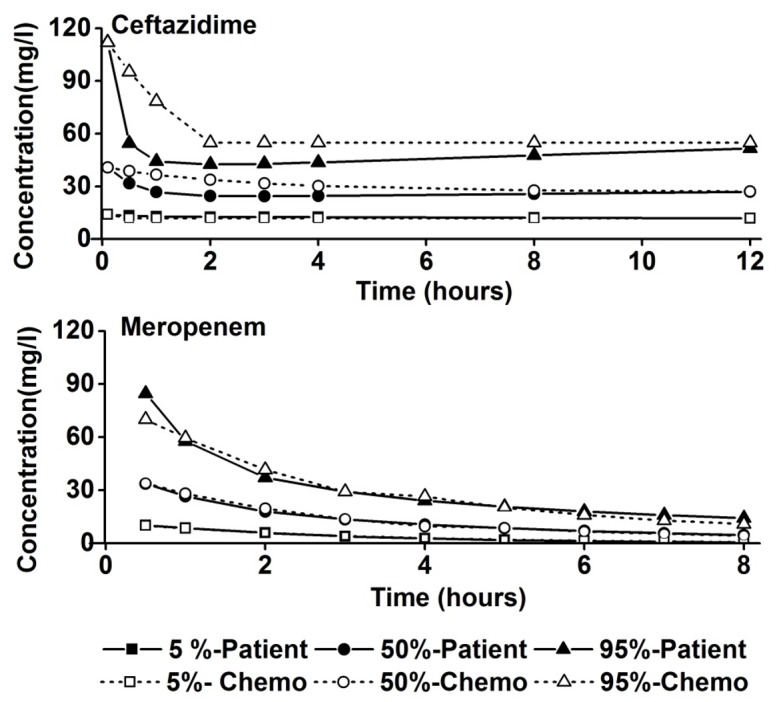
Comparison of the computer-simulated 5, 50, and 95 percentile concentrations of ceftazidime and meropenem as a function of time in critically ill patients and the concentrations achieved in the chemostat.

The 5, 50, and 95 percentile concentration-time profiles resulting from the computer-simulations, which are representative for the expected concentration-time profiles in critically ill patients, were mimicked for each antibiotic in the chemostat. The acquired concentration-time profiles in the chemostat matched with the simulated ceftazidime and meropenem concentration-time curves as intended [13,14] (Fig 1). There was considerable inter-individual variation in the concentrations calculated for patients, which is illustrated by the large difference between the 5 and 95 percentile values for both antibiotics. Both antibiotics showed a rapid drop in concentration after the initial loading dose, which could not be completely mimicked in the chemostat. Otherwise the simulated and the chemostat antibiotic concentrations were equivalent. The measured mutation prevention concentrations (MPC) were 48± 18.5 mg/l for ceftazidime and 16±9.2 mg/l for meropenem, which are approximately the 95 percentile concentrations of these two drugs.

**Fig 2:**
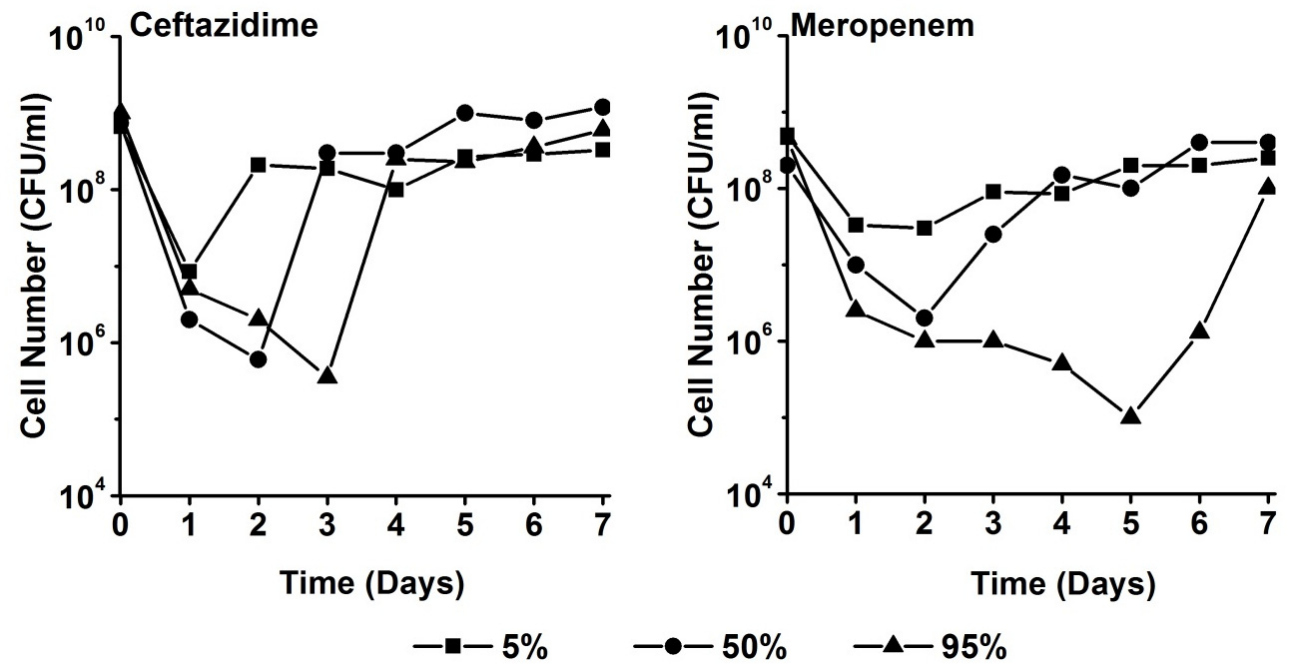
Density of a culture of *P*. *aeruginosa* exposed to ceftazidime and meropenem in the chemostat (D = 0.3 h^-1^) at concentrations simulating the 5, 50 and 95 percentile of the concentration-time profiles as they are expected in critically ill patients. Cultures were in steady state in the absence of antibiotics before day 0.

The bacterial cell density of the culture decreased by 2 to 4 factors of ten within the first 1–5 days (Fig 2). Decrease and recovery depended on the antibiotic concentrations applied. In all cases, the cultures almost completely returned to the initial density within maximally 7 days. The fastest recovery happened at the 5 percentile concentration of ceftazidime and the slowest upon exposure to the 95 percentile concentration of meropenem. In none of the experiments a wash-out occurred, as would be indicated by a complete disappearance of all bacterial cells. Instead, already after 24 hours the initial rapid decline slowed and some growth took place, though initially not always enough to completely counterbalance the dilution rate of 0.3 h^-1^.

**Fig 3:**
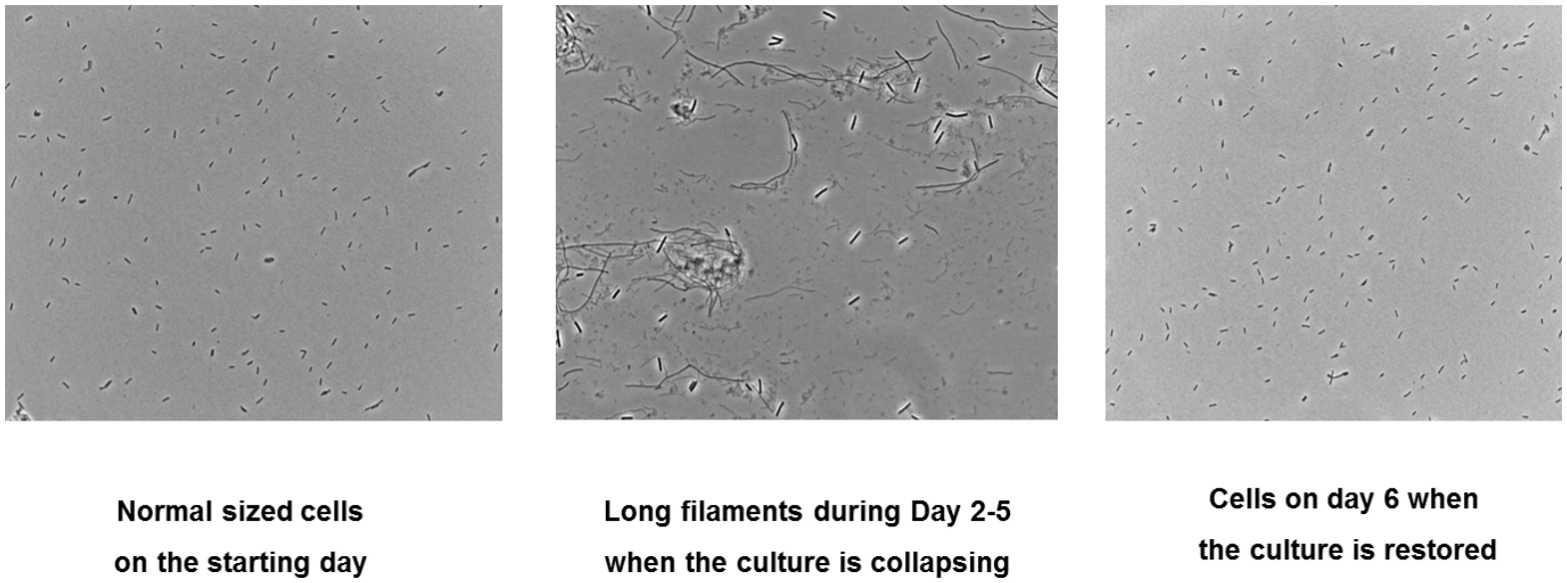
Comparison of cell morphology of regular growing culture and a culture growing at the 50 percentile concentration of ceftazidime during the initial exposure. Exposure to other concentrations of ceftazidime and all experimental concentrations of meropenem yielded a similar morphology.

Cell morphology was observed under the microscope at several stages during initial exposure to antibiotics and the subsequent recovery. In Fig 3, the morphology of a culture grown in the absence of ceftazidime is compared to that of a culture exposed to ceftazidime at the time when the cell density was lowest. Long filaments were seen when the culture was stressed upon initial exposure to ceftazidime. After the cultures recovered, the morphology was identical to that of the starting culture. The results at different concentrations were very similar and so was the morphology upon exposure to meropenem.

**Fig 4:**
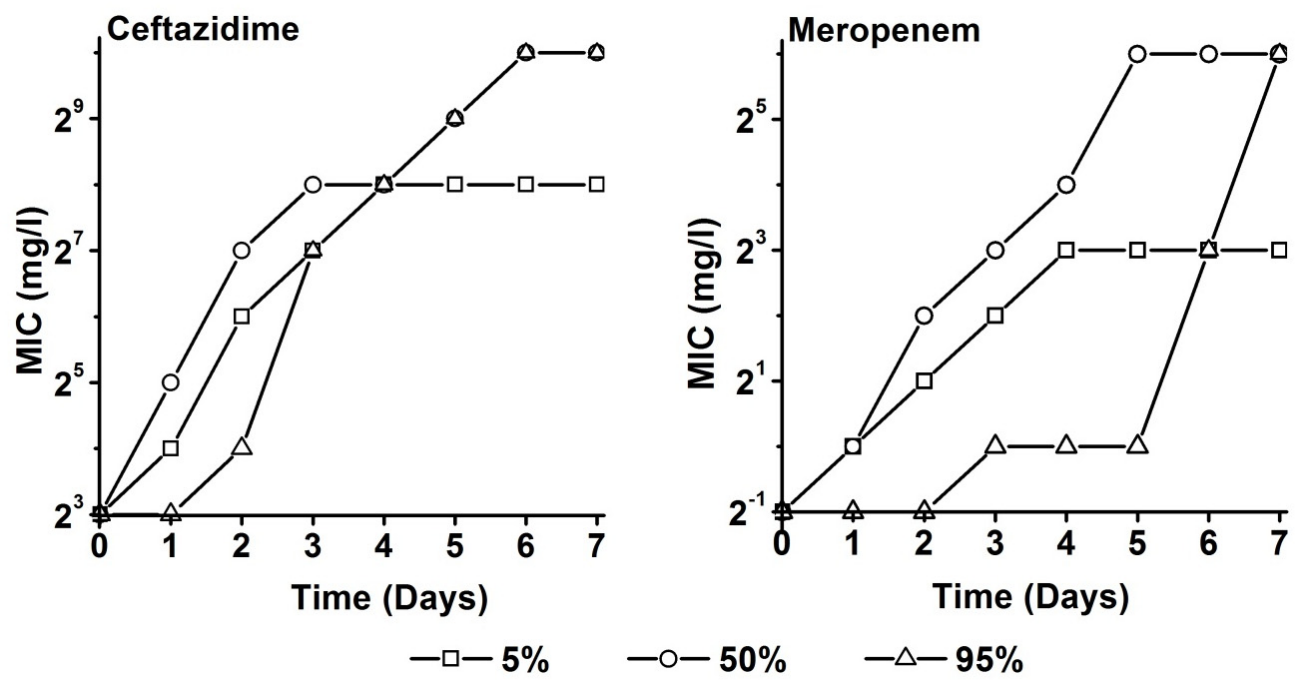
Minimum inhibitory concentrations (MIC) of *P*. *aeruginosa* culture as a function of time (days) during exposure to the 5, 50, and 95 percentile of concentration-time profiles as they are to be expected in critically ill patients of ceftazidime (left panel) and meropenem (right panel) in chemostats (D = 0.3 h^-1^).

The MIC increased by between 4 and 7 two-fold increasing steps during the length of the simulated treatment (Fig 4). For both antibiotics, the most rapid increase occurred at the median concentration. The 5 percentile concentration yielded a smaller and slower increase in MIC. The cells exposed to the highest drug level (95 percentile) were the last to show an increased MIC, however the final MIC values were the highest. After a continuously increasing phase, the MICs remained constant for all concentrations. The adaptation to meropenem seemed to require more time than to ceftazidime.

**Fig 5:**
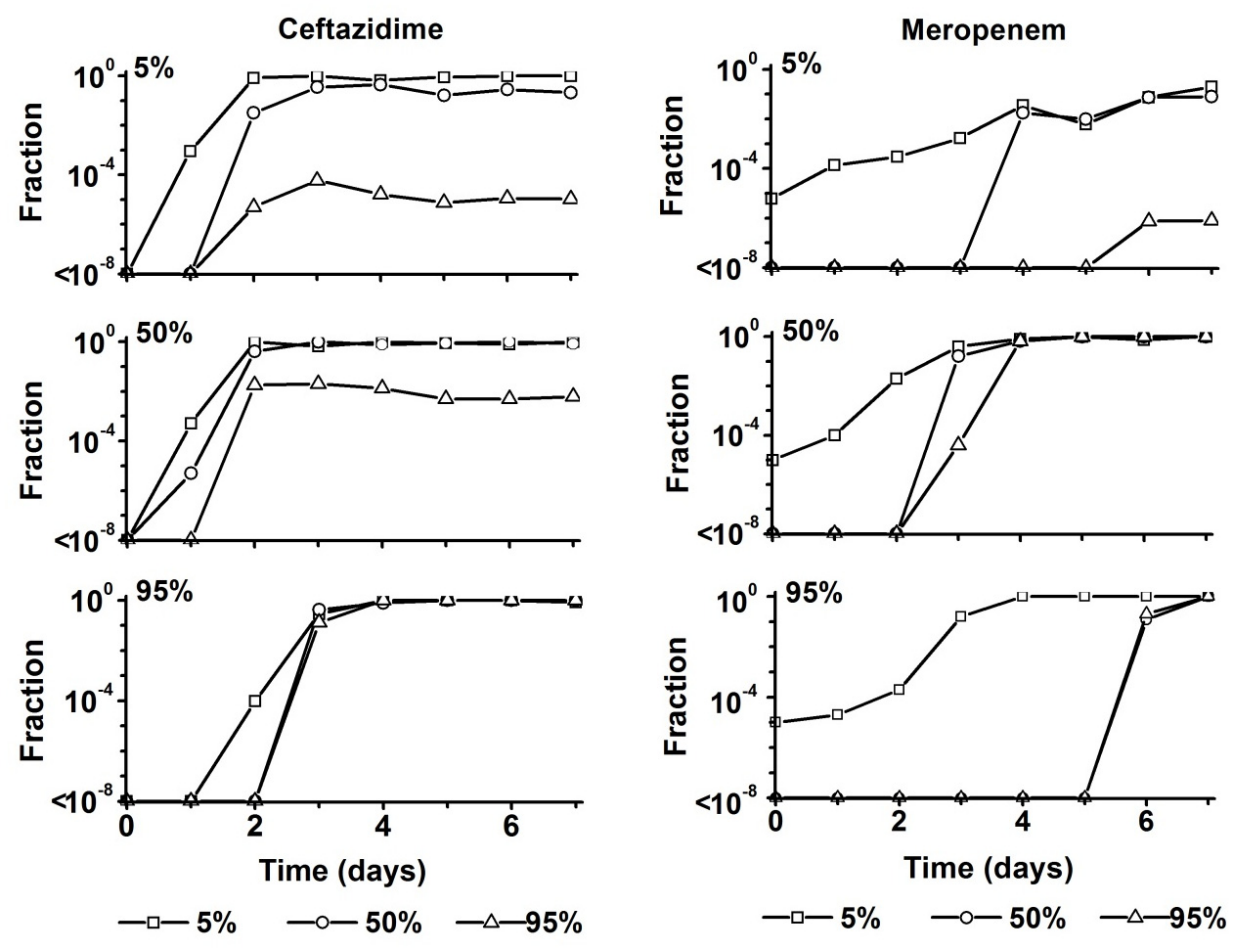
Fraction of cells from chemostat cultures that can grow on plates containing the 5, 50, 95 percentile of the ceftazidime steady-state concentration and the meropenem trough concentration (8 hour after drug administration) as these are to be expected in critically ill patients, as a function of time (days) during growth at the indicated antibiotic levels.

When resistance develops during antibiotic treatment of an infection, initially only a minute fraction of the total number of cells will be resistant. Still, the size and existence of this fraction is highly relevant as it forms the source for outgrowth of the resistant pathogens later on (Fig 5). The fraction of cells resistant to the 5, 50 and 95 percentile concentrations was determined by plating on agar plates containing these levels of each antibiotic. The largest fraction resistant to ceftazidime arose in the culture exposed to the 50 percentile level. Already after 2 days almost all cells were resistant at the 5 and 50 percentile concentrations and approximately 1% at the 95 percentile concentration. After exposure at the 5 percentile levels, only about 0.01% was resistant at the 95 percentile concentration, with no increase after 2 days. At the highest exposure, 95 percentile, it took longer for resistant cells to appear, but once that happened all cells were highly resistant. Exposure to meropenem yielded two major differences: At the start of the experiment a 10^−5^ fraction was resistant at the 5 percentile level, but the time it took to develop constant levels of resistance was much longer, up to 7 days.

**Fig 6:**
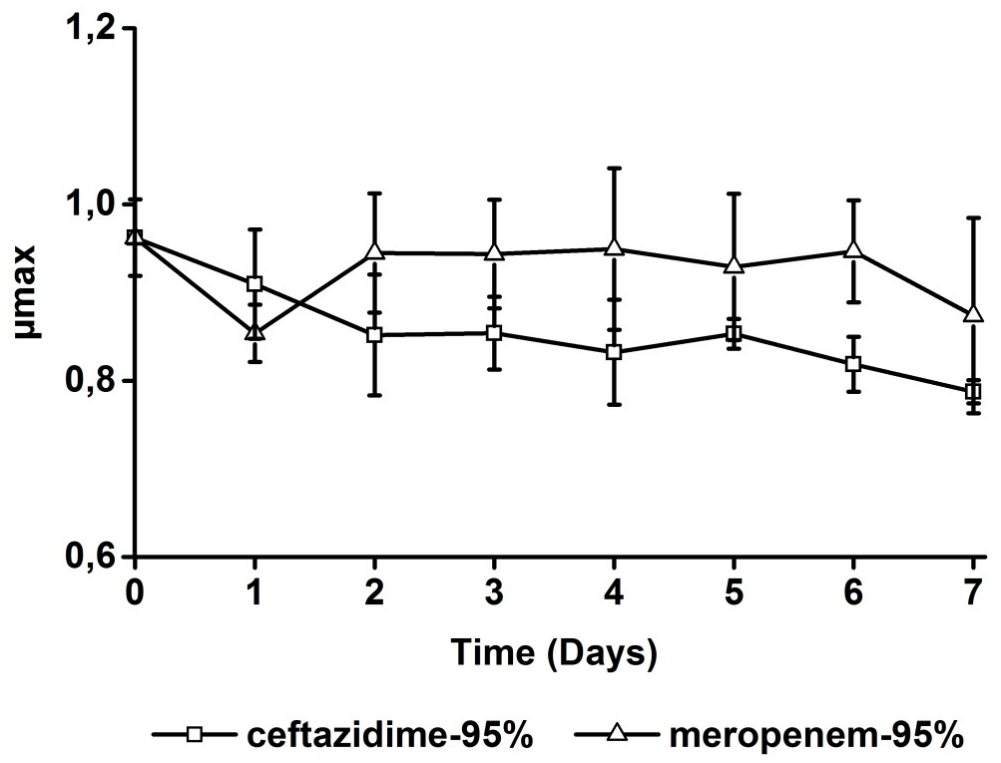
Maximum growth rate (μ_max_) in the absence of antibiotics of cells taken from chemostat cultures exposed to the 95 percentile of concentration-time profiles as they are to be expected in critically ill patients of ceftazidime or meropenem as a function of time (days). For each time point, the growth rate of four independent samples was measured.

The maximum growth rate (μ_max_) is often considered a measure for the relative fitness of bacteria. To examine whether the acquisition of resistance was accompanied by a loss of relative fitness, the μ_max_ in the absence of antibiotics was determined on samples taken from the chemostat cultures exposed to the highest level (95 percentile) of ceftazidime and meropenem. In response to growth at these drug levels, the μ_max_ decreased slightly in cells that became resistant to ceftazidime and decreased for only one day upon exposure to meropenem (Fig 6). The small difference suggests that relative cell fitness was barely affected during the evolution of drug resistance.

Contrary to the expectations based on whole genome sequencing of strains made resistant to very high concentrations of antibiotics by step-wise increasing exposure, the number of mutations detected after growth in the presence of ceftazidime and meropenem was very limited. Exposure to ceftazidime yielded two point mutations in ampD gene (Stop86E and V155G) (Table 2). In the case of meropenem several mutations were detected in *oprD*: Two point mutations (E107D, G166S), an insertion of C between position 389 and 390 and a deletion of A at position 296. In only one condition, exposure to the 95 percentile for 7 days, an insertion of G occurred between position 296 and 297 of *mexR*. Mutations occurred either relatively shortly after the start of the exposure, or only at the very end and only at the highest concentration, in the case of *mexR* and meropenem.

**Table 2:** Mutations detected in *P*. *aeruginosa* cultures during exposure to the 5, 50, and 95 percentile of concentration-time profiles as they are to be expected in critically ill patients of ceftazidime (*ampD*, *dacB*, *hfq*, *yerD*) and meropenem (*oprD*, *mexR*, *mexB*).

Time				Tested genes					
	*ampD*			*oprD*			*mexR*		
	5%	50%	95%	5%	50%	95%	5%	50%	95%
**D1**	√	⤫	⤫	⤫	⤫	⤫	⤫	⤫	⤫
**D2**	√	⤫	⤫	√	√	⤫	⤫	⤫	⤫
**D3**	√	√	√	√	√	⤫	⤫	⤫	⤫
**D4**	√	√	√	√	√	√	⤫	⤫	⤫
**D5**	-	-	-	-	-	-	-	-	⤫
**D6**	-	-	-	-	-	-	-	-	⤫
**D7**	√	√	√	√	√	√	⤫	⤫	√

Four and 14 randomly selected colonies were tested for meropenem and ceftazidime culture, respectively. No mutations were found in *dacB*, *hfq*, *yerD* and *mexB*.

## Discussion

The whole range of simulated antibiotic concentrations could induce the development of antibiotic resistance in slow-growing cultures of *P*. *aeruginosa* within 7 days, even at the highest drug levels. These results nicely dovetail with an earlier study that used shorter duration of exposure and less varied conditions [18]. It is therefore likely that similar processes occur during actual antibiotic therapy. Given the experimental conditions, these observations indicate that horizontal gene transfer is not required for development of resistance within a short time span during treatment. This seemingly contradicts, at least in the case of *P*. *aeruginosa*, the theory that high dosing is sufficient to avoid resistance development as the drug kills pathogenic bacteria before the bacteria have the chance to evolve drug resistance [7,11]. Apparently bacterial cells can survive and recover under conditions that do not allow normal growth. In any event, these observations may explain the occurrence of resistant infections shortly after high dose treatment was applied in the clinical setting [1].

The *de novo* development of antibiotic resistance is a complex interplay of initial changes in expression levels of a large number of genes and subsequent mutations in a few specific genes [5,8,9,19]. Some forms of resistance induce almost no fitness costs, hence a resistant mutant can often maintain itself once it emerges in a bacterial population [20]. Simulation of empirical treatment of infections by primary care physicians by exposing *Escherichia coli* to therapeutic concentrations of antibiotics during short time periods, resulted in resistance levels which could complicate subsequent treatment should this be necessary [21]. Protocols for treatment in the intensive care are explicitly designed to prevent this emergence of resistance [22]. In this study, however, exposure of *P*. *aeruginosa* to concentrations of ceftazidime and meropenem equal to those in the plasma of IC-patients for the usual treatment duration did allow resistant cells to develop and grow out and to finally dominate the culture. Combining the considerations discussed above, it seems that from the viewpoint of prevention of emergence of resistance, treatment with the highest dose the patient can endure for the shortest time that eliminates the infection, may be optimal.

The survival of 0.001%- 1% of cells at drug concentrations of 1 to 15 times the MIC observed in this study could possibly be attributed to drug resistance heterogeneity [23], collective resistance [24,25], the production of filaments [26,27], or a combination of these. According to the concept of resistance heterogeneity, a few cells among a population could temporarily tolerate higher drug concentrations by modifying cellular functions relevant to drug resistance, e.g. drug efflux, drug degrading enzymes, metabolic dormancy, etc. [8,28]. If the drug pressure is continued, these phenotypically resistant cells could acquire genetic mutations to achieve long lasting resistance [9]. The filamentation observed in this study at moments that the cells are under severe stress resulting from exposure to the antibiotics, is fully in line with the concepts described above.

The in some cases observed absence of cells able to grow at antibiotic concentrations encountered in the growth medium can be explained by assuming that the proportion of phenotypic resistant cells in the culture is below the detection limit of 10 cells/ml. According to the collective resistance theory [24,25], a small fraction of cells can escape the killing by lethal drug concentrations through co-operation among the susceptible cells by e.g. cooperative antibiotic inactivation, formation of biofilms, clustering, etc. Absence of resistant cells at the beginning of the treatment corresponds well with this explanation. The morphological changes seen in this study indicate that filamentation may be an essential step bacteria use to establish antibiotic resistance, in agreement with other studies [24,26,27]. Essential for this concept are the multiple chromosomes encompassed in a single filament. Recombination among these chromosomes on the one hand helps repairing DNA damage caused by antibiotic exposure, on the other hand it accelerates drug resistance evolution by increasing the mutagenesis rate [26]. Once the mutant chromosome is generated, it would be separated from the filament and propagate normal sized, resistant progeny.

The initial cell density in the chemostats of approximately 10^9^ CFU/ml was much larger than that in bacteremia (10^4−6^ CFU/ml), but comparable to what is found outside of the systemic circulation [29,30]. The cell number can increase to as much as 10^8−10^ CFU/mL in *Pseudomonas* biofilms, e.g. in sputum samples of cystic fibrosis patients [29,30]. Population size is one of the key factors influencing the evolution of antibiotic resistance [31]. Resistant subpopulations are present in low frequencies (i.e.10^-6^–10^−8^), so large population sizes will boost the rate at which bacteria can evolve [7]. In addition, collective resistance is expected to occur more readily at high cell density. The dependence of the evolutionary path on the selective power [32,33], may explain the observation that the time required for emergence of resistance increases and that the fraction of resistant cells decreases with increasing antibiotic concentration. This notion is further supported by the observation that at some concentrations cells resistant to different antibiotic levels were co-selected and their ratio maintained to the end.

Even at the highest tested concentrations as they are expected in patients, drug resistance will emerge and develop if treatments last long enough, underscoring the importance of limiting the length of treatments. The importance of short treatments is further stressed by the observation that the decrease of maximal growth rate of resistant cells is of short duration. Once cells have become resistant, the growth rate rapidly restores to rates comparable to those of susceptible cells. Hence an infection can be hardly cleared in subsequent treatments [34]. Once drugs kill the majority of the pathogenic bacteria, the immune system usually normally clears the left-over cells [35]. Therefore the survival of a small number of cells and the following development of resistance under adequate drug exposure will less likely happen in patients with competent immunity. Consequently, attention should be given to the design of the dosing regimen for patients with compromised immunity.

The overall conclusion of this study is that even the highest antibiotic concentrations measured in patients, although considerably exceeding the MIC, can still induce development of antibiotic resistance in a realistic treatment time. Induction of resistance occurs more rapidly at the lower concentrations than at the higher levels. The consequences are not only development of high levels of resistance but also recovery of fitness of resistant variants within the time frame of the treatment. This in turn might severely hamper a subsequent treatment, should this be necessary. Therefore, to prevent the occurrence of antibiotic resistance, strategies of high doses combined with the shortest treatment courses that is sufficiently effective, seem most effective.

## Supporting Information

S1 File Original data of graphs 3, 4 and 5. (XLSX)
